# From Bedsores to Bloodstream: A Rare Case of Elizabethkingia meningoseptica-Induced Sepsis

**DOI:** 10.7759/cureus.110356

**Published:** 2026-06-06

**Authors:** Malini Jagannatha Rao, Karthik Bhaskaran, Mohini Singh

**Affiliations:** 1 Department of Microbiology, Employees' State Insurance Post Graduate Institute of Medical Sciences and Research and Medical College, Bengaluru, IND; 2 Department of Microbiology, People's Education Society University (PESU) Institute of Medical Sciences and Research, Bengaluru, IND

**Keywords:** blood culture, elizabethkingia meningoseptica, gluteal bedsores, multidrug resistance, nosocomial pathogen, repeat culture, wound infection

## Abstract

*Elizabethkingia meningoseptica* (*E. meningoseptica*) is an emerging multidrug-resistant nosocomial pathogen that primarily affects debilitated and immunocompromised individuals. We report a rare case of *E. meningoseptica*-associated sepsis arising from chronic bilateral gluteal pressure ulcers in a 64-year-old bedridden male patient with diabetes mellitus, hypertension, and recurrent cerebrovascular accidents, highlighting the importance of considering uncommon pathogens in non-healing wounds. An initial wound culture yielded *Proteus mirabilis*, and the patient was started on intravenous meropenem. Despite therapy, there was no clinical improvement, with persistent wound discharge and progressive tissue necrosis. Repeat wound and blood cultures subsequently grew *E. meningoseptica*, which was identified using the VITEK 2 automated system (bioMérieux, Marcy-l'Étoile, France). Antimicrobial susceptibility testing performed using the VITEK 2 system demonstrated susceptibility to piperacillin-tazobactam, cefoperazone-sulbactam, ciprofloxacin, and cotrimoxazole, with resistance to carbapenems and aminoglycosides. Meropenem was discontinued, and targeted therapy with piperacillin-tazobactam was initiated. Significant clinical improvement was observed within five to seven days, with marked reduction in wound discharge and local inflammatory signs and progressive wound granulation by day 14. This case highlights the importance of repeat cultures, accurate microbiological identification, and close laboratory-clinician communication in patients with non-healing chronic wounds who fail to respond to empirical therapy.

## Introduction

*Elizabethkingia meningoseptica* (*E. meningoseptica*) is a Gram-negative, non-fermenting bacillus found in hospital water sources and medical equipment [1]. The organism was previously classified under the genus *Chryseobacterium *before being reclassified as *E. meningoseptica* [2]. Historically, *E. meningoseptica* has been recognized as a cause of neonatal meningitis and has increasingly been reported in healthcare-associated infections, including pneumonia, bacteremia, and device-associated infections, particularly among immunocompromised individuals and patients with prolonged hospital stays [1,3]. Reported mortality rates for *Elizabethkingia* bloodstream infections have ranged from approximately 20%-40% in critically ill patients, particularly in intensive care settings, reflecting their clinical severity and multidrug-resistant nature [1].

Clinically, this organism is important because it is associated with severe healthcare-associated infections and often exhibits resistance to multiple commonly used empirical antibiotics, leading to therapeutic failure if not promptly identified [4,5]. Outbreaks of *Elizabethkingia *species, including *Elizabethkingia anophelis*, have been reported in intensive care units (ICUs), highlighting its potential for nosocomial transmission [6]. Accurate laboratory identification of *Elizabethkingia *species remains challenging due to phenotypic similarity with other non-fermenting Gram-negative bacilli and the need for automated systems or molecular methods for reliable species-level differentiation.

Despite increasing recognition of *Elizabethkingia *species as nosocomial pathogens, there is limited published literature describing their clinical course, diagnostic challenges, and treatment outcomes in chronic pressure ulcer-associated infections, particularly in debilitated patients. This represents an important gap in understanding optimal diagnostic and therapeutic approaches for such atypical presentations.

We report a case of *E. meningoseptica* infection complicating bilateral gluteal pressure ulcers in a chronically ill, bedridden patient, with the aim of highlighting the diagnostic challenges, importance of repeat microbiological evaluation, and need for early targeted therapy in managing uncommon multidrug-resistant healthcare-associated infections.

## Case presentation

A 64-year-old male patient with a known history of type 2 diabetes mellitus, hypertension, and recurrent cerebrovascular accidents (three times in the past four years) presented with a foul-smelling wound, insidious onset, and gradually progressive over the gluteal region and lower back for 15 days. He had been bedridden for four years due to neurological deficits and required full-time nursing care. The patient was on antiplatelets, statins, antihypertensives, and oral hypoglycemics. He had no known drug allergies. Personal history revealed long-term smoking and alcohol use. There was no significant family history.

On admission, the patient was febrile with a temperature of 38.3°C, blood pressure of 100/60 mmHg, heart rate of 112 beats per minute, respiratory rate of 24 breaths per minute, and oxygen saturation of 96% on room air, consistent with systemic inflammatory response.

**Figure 1:**
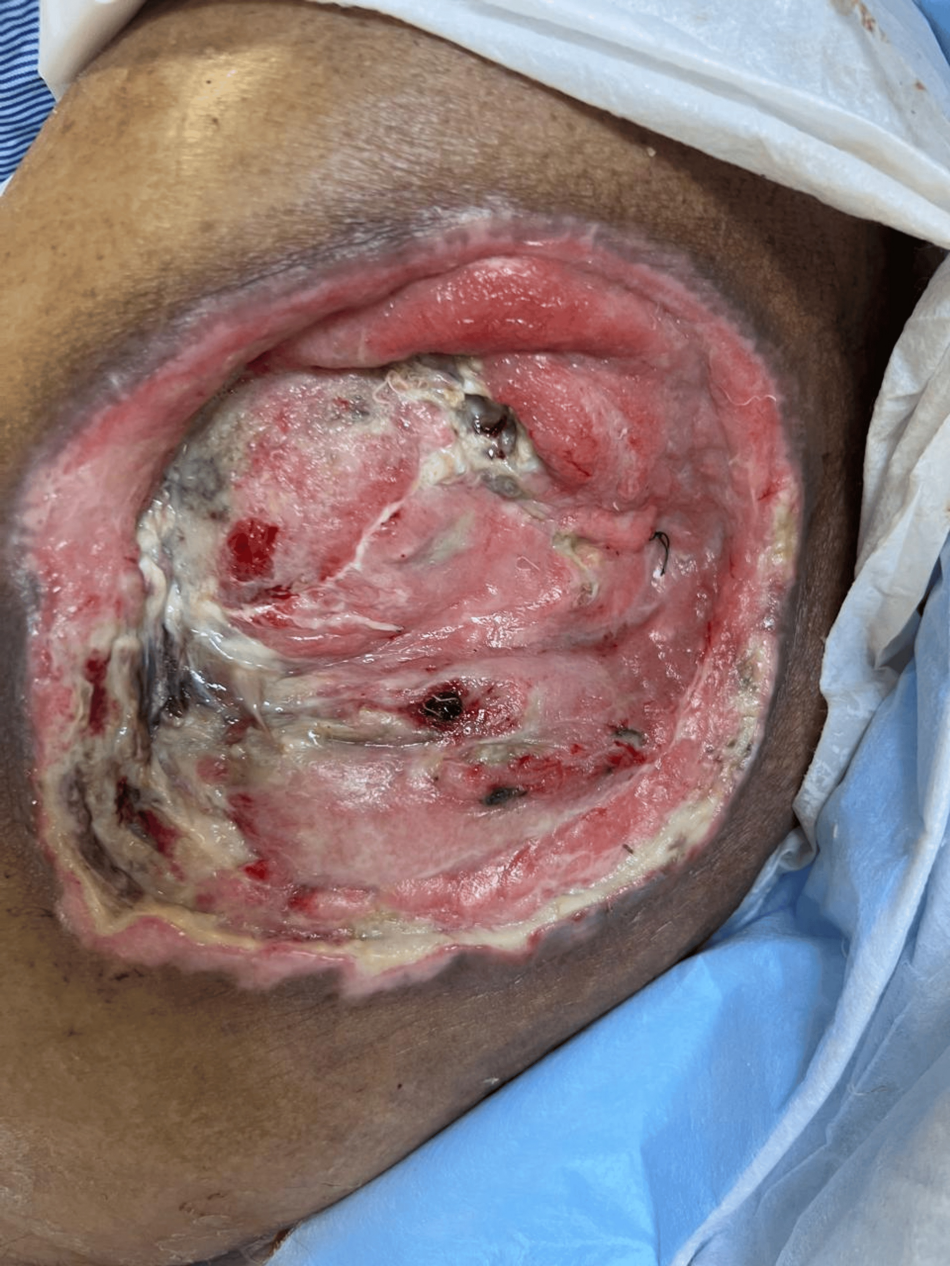
Bilateral gluteal pressure ulcers at presentation Bilateral Stage IV pressure ulcers over the gluteal region in the 64-year-old bedridden patient, showing extensive tissue loss with blackish necrotic slough and foul-smelling discharge. Findings are consistent with severely infected pressure injuries at admission.

Local examination showed extensive bilateral gluteal pressure ulcers with necrotic tissue and foul-smelling discharge. The right-sided ulcer measured 10 × 12 cm and the left 15 × 15 cm, with blackish discoloration, well-demarcated margins, and copious, foul-smelling, blood-tinged discharge (Figure 1). 

The pressure ulcers were classified as Stage IV, with extensive necrosis and deep tissue involvement involving bilateral gluteal regions. The clinical picture was consistent with clinical sepsis based on vitals and leukocytosis secondary to infected pressure ulcers.

On systemic examination, the patient had altered higher mental functions with bilateral lower limb power of 1/5. Cardiovascular, respiratory, and abdominal examinations did not reveal any significant abnormalities. Laboratory investigations at admission revealed a hemoglobin level of 7.1 g/dL (reference range: 13-17 g/dL) and a total leukocyte count of 19,170 cells/mm³ (reference range: 4,000-11,000 cells/mm³). Renal function tests were within normal limits, while liver function tests were deranged.

Based on the initial isolation of *Proteus mirabilis *from the wound swab, the patient was started on intravenous meropenem 500 mg twice daily. Serial inflammatory markers showed persistent leukocytosis with no clinical improvement, with persistent discharge and progressive tissue necrosis during initial empirical therapy.

Microbiological findings

Due to the lack of clinical response, repeat wound swabs and blood cultures were obtained. On culture, MacConkey’s agar showed no growth, and blood agar showed non-hemolytic grey colonies. Both cultures yielded *E. meningoseptica*, identified using the VITEK 2 automated identification system (bioMérieux, Marcy-l'Étoile, France). Antimicrobial susceptibility testing was performed using the VITEK 2 system, and results were interpreted according to Clinical and Laboratory Standards Institute (CLSI) guidelines. The isolate showed sensitivity to piperacillin-tazobactam, cefoperazone-sulbactam, ciprofloxacin, and cotrimoxazole and resistance to carbapenems and aminoglycosides (Table 1). 

**Table 1:** Antimicrobial susceptibility profile of Elizabethkingia meningoseptica isolate (VITEK® 2, interpreted using CLSI guidelines) MIC: minimum inhibitory concentration; S: sensitive;  R: resistant; CLSI: Clinical and Laboratory Standards Institute

Isolate: *Elizabethkingia meningoseptica*		
Antimicrobial agent	MIC	Interpretation
Piperacillin–tazobactam	8	S
Ceftazidime	1	S
Cefoperazone–sulbactam	≤8	S
Aztreonam	8	S
Imipenem	≥16	R
Meropenem	≥16	R
Amikacin	≥64	R
Ciprofloxacin	0.5	S
Trimethoprim/Sulfamethoxazole	<20	S

No imaging studies were performed as the diagnosis was clinically evident and supported by microbiological findings.

Treatment and follow-up

Following repeat cultures isolating *E. meningoseptica* from both wound and blood samples, antimicrobial therapy was modified according to susceptibility results. Meropenem was discontinued on day 6, and intravenous piperacillin-tazobactam 4.5 g twice daily was initiated. Alternative agents such as fluoroquinolones and trimethoprim-sulfamethoxazole were considered; however, piperacillin-tazobactam was selected based on in vitro susceptibility and clinical context. Supportive wound management included regular wound cleansing, serial surgical debridement of necrotic tissue, pressure off-loading measures, and routine dressing changes. Clinical improvement was observed within five to seven days of therapy modification. The identification of *E. meningoseptica*, a pathogen intrinsically resistant to carbapenems [4, 5], was a turning point in management. Wound discharge decreased markedly, local inflammatory signs subsided, and the patient's overall clinical condition improved (Table 2).

**Table 2:** Clinical timeline, microbiological findings, antimicrobial therapy, and treatment response in a patient with Elizabethkingia meningoseptica–associated bilateral gluteal pressure ulcers BD: twice daily; CLSI: Clinical and Laboratory Standards Institute; AST: antimicrobial susceptibility testing

Day/Time	Clinical Event	Microbiology	Treatment	Outcome
Day 0	Admission with bilateral Stage IV pressure ulcers	Wound culture: *Proteus mirabilis*	Meropenem 500 mg IV BD started	No improvement
Day 5	Persistent sepsis and wound deterioration	Repeat wound + blood culture sent	Continued meropenem	No response
Day 6–7	Identification of *Elizabethkingia meningoseptica*	VITEK 2 identification, CLSI AST	Switched to piperacillin-tazobactam 4.5 g IV BD	Clinical improvement
Day 14	Wound improvement	—	Continued targeted therapy + debridement	Granulation tissue seen

**Figure 2:**
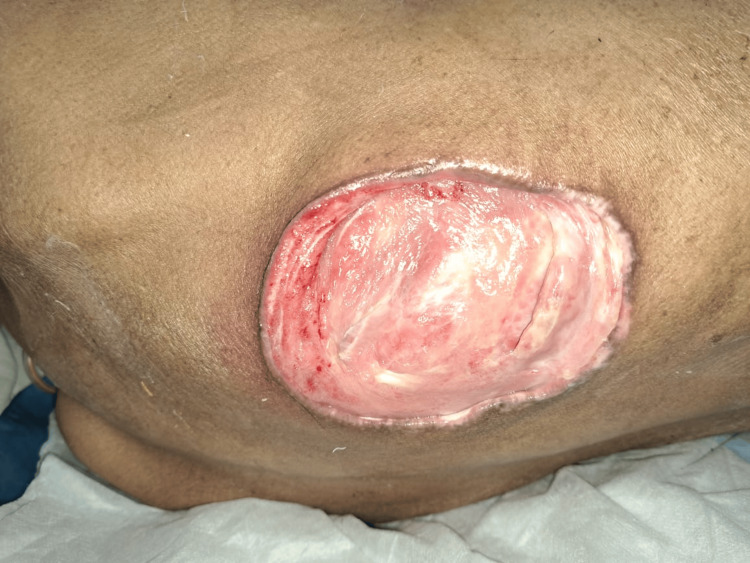
Post-treatment improvement of pressure ulcers Post-treatment image showing reduction in necrotic tissue and exudate following targeted antimicrobial therapy and serial debridement. Early granulation tissue formation and decreased inflammatory changes are evident.

Continued wound care and serial debridement facilitated progressive healing, with healthy granulation tissue evident by day 14 (Figure 2).

## Discussion

*E. meningoseptica* is an emerging opportunistic, non-fermenting Gram-negative bacillus that has been increasingly reported as a cause of healthcare-associated infections [1,4]. It is commonly found in hospital water systems, moist environmental surfaces, and medical equipment, reflecting its ability to persist in low-nutrient environments. The organism’s capacity for biofilm formation contributes significantly to its survival in hospital settings and its potential for nosocomial transmission. In addition, its intrinsic resistance to multiple antibiotic classes, including carbapenems and aminoglycosides, poses significant therapeutic challenges and often leads to delayed or inappropriate empirical treatment.

Historically, *E. meningoseptica* has been associated with neonatal meningitis and outbreaks in ICUs, particularly among immunocompromised and critically ill patients. More recently, it has been increasingly recognized as a cause of bacteremia and device-associated infections in hospitalized adults. However, its isolation from chronic pressure ulcers remains extremely rare, with limited literature describing soft tissue involvement. The present case adds to this growing body of evidence by highlighting its role as a potential pathogen in chronic, non-healing pressure sores in a debilitated patient [7].

The initial isolation of *Proteus mirabilis *from the wound culture likely represented either polymicrobial colonization or a superficial wound pathogen, as there was no clinical response to targeted carbapenem therapy. In this case, isolation of *E. meningoseptica* from both blood and wound cultures in a clinically deteriorating patient, along with a rapid response to targeted therapy, supports its role as a true pathogen rather than a colonizer.

Clinically, persistent non-healing wounds that fail to respond to broad-spectrum empirical therapy, particularly carbapenems, should raise suspicion for uncommon non-fermenting Gram-negative bacilli such as *Elizabethkingia *spp. Early consideration of such pathogens is essential, as inappropriate empirical therapy may delay targeted treatment and worsen outcomes [7]. The present case also emphasizes the importance of repeated microbiological evaluation in the setting of clinical non-response, which ultimately led to the identification of the causative organism and appropriate antimicrobial modification.

Limitations

This report has certain limitations. As a single-patient case report, the findings cannot be generalized. Molecular characterization or genomic sequencing of the isolate was not performed, limiting detailed understanding of strain-relatedness and potential environmental sources. Additionally, environmental sampling of hospital water systems was not undertaken to identify possible reservoirs of infection. The follow-up period was limited to the short-term clinical course, and long-term outcomes after discharge could not be assessed.

Future directions

Future studies should focus on systematic surveillance of hospital water systems and medical equipment to identify potential reservoirs of *Elizabethkingia *species. Further research is also required to better understand optimal antimicrobial regimens, including the role of combination therapy and biofilm-targeted strategies, particularly in chronic wound infections. Strengthening infection control practices and antimicrobial stewardship programs will be essential in preventing and managing such emerging nosocomial pathogens.

## Conclusions

*E. meningoseptica* is an uncommon but increasingly recognized healthcare-associated pathogen with intrinsic resistance to multiple antimicrobial agents. This case highlights the importance of considering uncommon multidrug-resistant organisms in patients with chronic non-healing wounds who fail to respond to empirical therapy. Repeat microbiological evaluation, accurate laboratory identification, and close clinician-microbiologist communication facilitated targeted antimicrobial therapy and were associated with favorable clinical improvement in this patient. Increasing reports of *Elizabethkingia *infections in the literature underscore the need for continued clinical awareness and timely microbiological diagnosis when managing similar presentations.
